# Milk-Fat-Globule-Membrane-Enriched Dairy Milk Compared with a Soy-Lecithin-Enriched Beverage Did Not Adversely Affect Endotoxemia or Biomarkers of Gut Barrier Function and Cardiometabolic Risk in Adults with Metabolic Syndrome: A Randomized Controlled Crossover Trial

**DOI:** 10.3390/nu15143259

**Published:** 2023-07-23

**Authors:** Avinash Pokala, William R. Quarles, Joana Ortega-Anaya, Rafael Jimenez-Flores, Sisi Cao, Min Zeng, Joanna K. Hodges, Richard S. Bruno

**Affiliations:** 1 Human Nutrition Program, The Ohio State University, Columbus, OH 43201, USA; pokala.2@osu.edu (A.P.); williamrquarles@gmail.com (W.R.Q.); caosisi0703@gmail.com (S.C.); zeng.670@osu.edu (M.Z.); jku103@psu.edu (J.K.H.); 2 Department of Food Science and Technology, The Ohio State University, Columbus, OH 43201, USA; joana_oa@outlook.com (J.O.-A.); jimenez-flores.1@osu.edu (R.J.-F.); 3 Department of Nutritional Sciences, The Pennsylvania State University, State College, PA 16802, USA

**Keywords:** milk fat globule membrane, metabolic syndrome, cardiometabolic health, endotoxin

## Abstract

Full-fat dairy milk may protect against cardiometabolic disorders, due to the milk fat globule membrane (MFGM), through anti-inflammatory and gut-health-promoting activities. We hypothesized that a MFGM-enriched milk beverage (MEB) would alleviate metabolic endotoxemia in metabolic syndrome (MetS) persons by improving gut barrier function and glucose tolerance. In a randomized crossover trial, MetS persons consumed for two-week period a controlled diet with MEB (2.3 g/d milk phospholipids) or a comparator beverage (COMP) formulated with soy phospholipid and palm/coconut oil. They then provided fasting blood and completed a high-fat/high-carbohydrate test meal challenge for evaluating postprandial metabolism and intestinal permeability. Participants had no adverse effects and achieved high compliance, and there were no between-trial differences in dietary intakes. Compared with COMP, fasting endotoxin, glucose, incretins, and triglyceride were unaffected by MEB. The meal challenge increased postprandial endotoxin, triglyceride, and incretins, but were unaffected by MEB. Insulin sensitivity; fecal calprotectin, myeloperoxidase, and short-chain fatty acids; and small intestinal and colonic permeability were also unaffected by MEB. This short-term study demonstrates that controlled administration of MEB in MetS persons does not affect gut barrier function, glucose tolerance, and other cardiometabolic health biomarkers, which contradicts observational evidence that full-fat milk heightens cardiometabolic risk. Registered at ClinicalTrials.gov (NCT03860584).

## 1. Introduction

Metabolic syndrome (MetS) afflicts ≈35% of American adults, and its prevalence increases to ≈50% among those >60 years of age [1]. This cardiometabolic disorder is characterized by having any three of five risk factors, specifically hyperglycemia, hypertension, hypertriglyceridemia, increased waist circumference, and depressed high-density lipoprotein-cholesterol. Tragically, individuals with MetS experience a two-times higher risk of premature mortality due to cardiovascular disease (CVD), type 2 diabetes, and other cardiometabolic conditions [2]. Because most MetS persons are subclinical such that pharmacological management is contraindicated, establishing effective lifestyle interventions that can manage MetS remains critical.

Consistent with increased circulating endotoxin concentrations in MetS persons [3], metabolic endotoxemia has been suggested to initiate and exacerbate inflammatory responses that drive cardiometabolic complications, including the development of hyperglycemia [4]. Indeed, increased intestinal permeability permits translocation of gut-derived endotoxins (e.g., lipopolysaccharides (LPS) from Gram-negative bacteria) into the systemic circulation [5]. Consequently, LPS binds to Toll-like receptor-4 to activate NFκB-dependent inflammation (i.e., TNFα) [5], which mediates insulin resistance and related metabolic derangements [4]. While numerous factors contribute to gut barrier dysfunction, a Western diet has been implicated [6]. In particular, poor diet quality drives hyperglycemia [7], which damages tight junctions of the intestinal epithelium resulting in ‘leaky gut’ [8]. Thus, nutrition strategies that improve gut barrier function would be expected to alleviate endotoxemia-associated inflammation and cardiometabolic risk.

Increased consumption of bovine dairy foods could be an effective nutrition strategy to alleviate MetS-related complications. Evidence from retrospective and prospective observational studies suggests a 14–17% lower risk of developing MetS among those with higher intakes of dairy [9]. Others also report that dairy milk consumption protects against age-related increases in blood pressure and improves cardiovascular function [10]. The nutrient-rich matrix of dairy milk likely contributes to these health benefits, consistent with calcium being associated with a reduction in cardiovascular morbidity [11] and whey protein improving endothelial function, glycemic control, and lipid biomarkers [12,13]. Epidemiological studies also suggest that the fat content of dairy milk provides cardioprotective bioactivities. For example, a large-scale prospective observational study (*n* = 112,922) revealed a 28% lower risk of developing MetS among those who consumed >2 vs. 0 dairy servings of full-fat dairy products [14]. As we reviewed [15], benefits of dairy fat are potentially attributed to milk fat globule membrane (MFGM), a unique tri-layer membrane of polar lipids and proteins that encapsulate neutral lipids of milk fat. Importantly, MFGM is more greatly enriched in full-fat dairy milk compared with lower-fat varieties.

MFGM has received extensive attention as a component in infant formula [16]. However, its health-promoting bioactivities have been studied limitedly in metabolically compromised adults. This is despite pre-clinical evidence suggesting that its polar lipid fraction (i.e., phospholipids and sphingolipids) alleviates hyperlipidemia [17] and its membrane-bound proteins/enzymes protect against inflammation [18]. Furthermore, MFGM itself protects against gut barrier dysfunction and premature mortality in LPS-challenged mice [19]. MFGM also provides prebiotic activities on the gut microbiome to reduce pyrogenic Gram-negative bacteria, promote enrichment of commensal bacteria, and increase short-chain fatty acids (SCFAs, e.g., butyrate) that maintain gut barrier integrity [17,20]. Thus, we hypothesized that an MFGM-enriched beverage (MEB) compared with a matched comparator beverage enriched with soy phospholipids (COMP) would decrease metabolic endotoxemia in MetS persons by improving glucose tolerance and decreasing intestinal permeability and inflammation. To test this, we conducted a two-week double-blind, randomized crossover trial in which a controlled eucaloric diet was administered prior to assessments of metabolic endotoxemia, systemic and gut inflammation, and associated cardiometabolic and gut barrier function biomarkers.

## 2. Materials and Methods

Complete details of this double-blind, randomized controlled trial, including its rationale, methodology, and expected outcomes, have been reported previously [21]. All procedures were approved by The Ohio State University (OSU) Institutional Review Board (2018H0564), and the study was registered at ClinicalTrials.gov (NCT03860584).

### 2.1. Participant Eligibility

Adult men and women with MetS were recruited from the Columbus, OH, area from August 2019 to December 2020, but with a pause in all study activities from March 2020 through August 2020 due to the COVID-19 pandemic that temporarily halted all research at OSU. Participants provided written informed consent before performing any study procedures. Study eligibility was based primarily on age (18–65 y) and being classified with MetS by meeting ≥3 of the 5 established criteria [22]: waist circumference ≥ 102 cm in males or ≥88 cm in females; plasma triglyceride ≥ 150 mg/dL; plasma HDL-C < 40 mg/dL in males or <50 mg/dL in females; systolic blood pressure ≥ 130 mmHg or diastolic blood pressure ≥ 85 mmHg; and fasting glucose ≥ 100 mg/dL. Waist circumference was determined at the level of the umbilicus, and blood pressure was reported as the mean of two measures taken 5 min apart. Persons were ineligible if any of these criteria existed: dietary restrictions (e.g., vegetarian), food allergies (e.g., lactose or gluten intolerance), or were unwilling to consume prescribed eucaloric diets; consuming > 2 alcoholic drinks daily; using any medications that affect blood glucose, blood lipids, or blood pressure; using any antibiotics, anti-inflammatory agents, or probiotic supplements within the past 1 month; unstable body mass (±2 kg) during the past 3 months; or any history of liver or cardiovascular disease, cancer, or gastrointestinal disorders.

### 2.2. Study Design

Eligible participants were randomized to complete the double-blind crossover trial in which they were provided a eucaloric diet for 2 weeks that contained 3 daily servings (250 mL/serving) of a specially formulated bovine dairy milk beverage that was enriched with MFGM (MEB) or a comparator beverage (COMP) that was matched in macronutrients and prepared with soy phospholipids (lecithin). The treatment order was assigned by simple randomization generated by R software (R 3.6.3). Each two-week treatment period was separated by a two-week washout to test the hypothesis that MEB compared with COMP would decrease serum endotoxin (primary outcome) by improving gut barrier function.

An intervention period of 2 weeks was selected to evaluate the anti-inflammatory activities of MEB without the potential confounding effects of weight loss, which is more likely to occur in a longer-term study [23] and is known to affect endotoxemia [24]. In addition, our previous work demonstrates 2 weeks of a controlled diet in persons with MetS was sufficient to alleviate postprandial endotoxemia in response to a glucose challenge. During each study arm, participants visited the study center every 3–4 days to provide spot urine samples for compliance assessment and to obtain prescribed foods. Participants’ anthropometrics and blood pressure were assessed on days 0 and 13. Fasting blood samples were collected from the antecubital vein on day 0 and day 13, and at 30 min intervals for 180 min on day 13 from an in-dwelling catheter after ingesting a high-fat/high-carbohydrate test meal. Whole blood was collected into blood tubes containing no anticoagulants to obtain serum or tubes containing ethylenediaminetetraacetic acid (EDTA) or sodium heparin to obtain plasma. Serum and plasma were isolated from whole blood following centrifugation (3000× *g*, 15 min, 4 °C). An aliquot of EDTA-treated whole blood was also mixed with RNAlater^TM^ (ThermoFisher Scientific, AM 7020, Waltham, MA, USA) on day 0 and day 13, and at 180 min post-test meal ingestion to preserve RNA for future measures of pro-inflammatory gene expression.

On day 13, a high-fat/high-carbohydrate test meal was administered to induce acute endotoxemia [25] and permit the assessment of postprandial metabolic excursions. It consisted of 3 slices of white bread, 100 g of margarine, and a 75 g glucose solution (ThermoFisher Scientific) that contained non-digestible sugars (1 g sucralose, 5 g lactulose, 1 g mannitol, 1 g erythritol) to assess gut permeability (described below). The test meal provided 1070 kcal with 66% of energy from fat and 33% from carbohydrate. Participants also collected a stool sample during the preceding 24 h of their day 13 study visit using a commode specimen collection system (ThermoFisher Scientific). Stool samples were weighed and characterized according to the Bristol stool scale [26]. A complete 24 h urine sample was obtained from 0 to 5 h and 5 to 24 h at the end of each study arm; a 10% thymol solution was added to each urine collection container to inhibit bacterial growth. All biospecimens were aliquoted into sterile cryovials, flash-frozen in liquid nitrogen, and stored at −80 °C until analyzed. Upon completion of these procedures, participants underwent at least a two-week washout period before repeating the same study procedures but with allocation to the alternative test beverage.

### 2.3. Test Beverage Formulation and Compliance to Test Beverages

MEB and COMP beverages were prepared in the OSU Food Pilot Plant under Good Manufacturing Practices in 50 L batches. Beverages were pasteurized, stored at 4 °C in single-serve 250 mL bottles, and formulated to match the composition of full-fat bovine dairy milk (12.5% total solids, 3.3% of total protein, 4.8% lactose, and 3.5% total fat). MEB contained 10% MFGM phospholipid ingredient (Lipid 100, Fonterra Co-operative Group, Auckland, New Zealand), 40% butter oil (NutraPro International, Logan, UT, USA), 50% nonfat dairy milk powder (Dairy America, Dublin, CA, USA), and water to yield a total fat content of 3.5% (*w*/*v*). COMP contained 10% soy lecithin granules (General Nutrition Corporation, Pittsburgh, PA, USA), 40% palm oil/coconut oil (75:25; Columbus Vegetable Oils, Des Plaines, IL, USA), 50% nonfat dairy milk powder, and water to yield a final fat content of 3.5% (*w*/*v*). Both beverages had trace amounts of vanilla extract added to achieve study blinding by masking the subtle ‘beany’ flavor of soy lecithin in COMP.

Our formulation goal was to produce an MFGM-enriched milk product comparable to commercial full-fat dairy milk, except it contained 10-times more MFGM phospholipids [27]. MEB (3 daily servings, 8 oz each) contained 2.3 g total phospholipid (Table 1), which is consistent with total phospholipid intakes (2–8 g/d) of the Western diet [28] while recognizing that polar lipids are also derived from non-dairy products (e.g., egg yolk, peanuts). COMP was formulated in a near-identical manner except that its phospholipid content was soy lecithin, and a blend of plant oils provided the neutral lipid content. Both test beverages had similar total fat, saturated fat, and unsaturated fat (Table 1). Importantly, both beverages had equivalent total phospholipid content, but their individual species and distribution differed. MEB contained higher amounts of sphingomyelin, phosphatidylserine, and phosphatidylethanolamine, consistent with their proportional composition in MFGM, and lower amounts of phosphatidylinositol and phosphatidylcholine compared with soy-lecithin-containing COMP.

MEB and COMP also contained 100 mg Potaba^®^ (78% para-aminobenzoic acid, PABA; Glenwood LLC, Englewood, NJ, USA) per 250 mL serving to objectively evaluate compliance to test beverage consumption based on urinary PABA excretion. Our approach is consistent with others [29], which recognizes that dietary PABA exposure is low and that nearly 100% is eliminated in the urine within 24 h [30]. In brief, PABA was assessed from spot urine samples collected throughout each study arm, as described [30]. Samples were mixed with sodium hydroxide and incubated (100 °C, 1 h) prior to mixing with hydrochloric acid. This generates a diazonium salt that reacts with ammonium sulfamate and *N*-(1-naphthyl) ethylenediamine dihydrochloride to produce a chromophore that can be quantified spectrophotometrically at 540 nm against standards prepared in parallel. Compliance was defined as urinary PABA > 30 mg/L based on evidence that this threshold is ≥3 times greater than usual dietary exposures to PABA [30].

### 2.4. Dietary Control and Assessment

Participants were provided identical controlled diets during each intervention period, except for the assigned test beverage. Daily energy requirements were calculated using the Harris–Benedict equation [31] with adjustment for physical activity. A four-day rotating menu (Table S1) was used to formulate controlled diets at four different energy levels, achieved by modifying food portion sizes, ranging from 2200 to 3100 kcal/day in 200–300 kcal increments. Macronutrient distribution of diets was 54–55% carbohydrate, 28–29% fat, and 17–18% protein. Participants were assigned a daily energy level that provided minor excess to ensure sufficient food and to minimize deviations from the prescribed diet. The controlled diet was devoid of any dairy foods, fermented foods, and probiotic-containing foods. Dietary fiber was also restricted to 7.8 g/1000 kcal to match the typical American diet that is low in fiber (≈16 g/d) [32] and best examine the potential gut-level benefits of MEB. Table S2 shows the nutrient composition of the four-day 2200 kcal diet.

Participants were instructed to record any deviations from the prescribed diet in a provided food journal. All empty food containers and/or uneaten prescribed foods were returned to the study center to calculate actual food consumption. Prescribed and non-prescribed foods were entered into Nutrition Data System for Research dietary analysis software (NDSR 2022, University of Minnesota, Minneapolis, MN, USA) to determine energy and nutrient intakes and the proportion of energy intake from non-prescribed foods. NDSR was also used to determine participants’ Healthy Eating Index (HEI-2015) [33] to evaluate between-trial compliance to prescribed diets based on the composite score of the dietary pattern. HEI-2015 scores did not include the test beverage to limit the analysis to only the basal diet.

### 2.5. Metabolic Health

Plasma glucose (G75171L) and lipids (total cholesterol, C75101L; HDL-C, H751160; triglyceride, T7532500) were measured on a Synergy H1 microplate reader (Bio-Tek, Washington, DC, USA) using separate clinical assays (Pointe Scientific, Canton, MI, USA). Intra- and inter-assay coefficient of variations (CV) were 1.1–5.5% and 2.7–8.0%, respectively. LDL-cholesterol was calculated according to the Friedewald equation [34]. Plasma insulin (ALPCO (Salem, NH, USA), 80-INSHU-E01.1), glucagon-like peptide-1 (GLP-1; EMD Millipore (Burlington, MA, USA), EGLP-35K), and gastric inhibitory polypeptide (GIP; EMD Millipore, EZHGIP-45K) were measured by ELISA according to the manufacturer’s instructions (intra- and inter-assay CV = 3.0–4.9% and 7.3–14.0%, respectively). The homeostatic model assessment for insulin resistance (HOMA-IR) was calculated from fasting glucose and insulin as described [35]. Whole-body insulin sensitivity was calculated according to the Matsuda–DeFronzo Insulin Sensitivity Index [36] using postprandial glucose and insulin concentrations obtained during the 3 h test meal challenge.

### 2.6. Endotoxemia

Serum endotoxin was measured using a fluorometric PyroGene™ recombinant factor C assay (Lonza (Basel, Switzerland), 50-658U). The assay is based on endotoxin-mediated activation of recombinant factor C, which then cleaves a fluorogenic compound that can be monitored at 380/440 nm (excitation/emission). In brief, serum was diluted 1:100 in endotoxin-free water, incubated (10 min, 70 °C), and mixed with kit reagent prior to monitoring fluorescence at 0 min and 60 min following incubation (37 °C). Quantification was based on the net fluorescence of samples against that of endotoxin standards prepared in parallel. Intra- and inter-assay CVs were 2.6% and 10.6%, respectively. Plasma LPS-binding protein (LBP; DY870) and soluble CD14 (sCD14; DC140) were measured using separate ELISA kits (R&D Systems, Minneapolis, MN, USA) as secondary measures of endotoxin exposure [37]; intra- and inter-assay CVs were 2.2–7.2% and 6.2–24.5%, respectively.

### 2.7. Systemic and Intestinal Inflammation

Intestinal inflammation was assessed based on measures of fecal calprotectin (30-CALPHU-CH01) and myeloperoxidase (30-6630; Alpco). Assays were performed in accordance with the manufacturer’s instructions, and an easy stool extraction device (30-EZEX-100) was used to accurately measure stool mass prior to sample analysis. Intra- and inter-assay CVs of both proteins were <3.0% and <7.3%, respectively.

Whole blood pro-inflammatory gene expression was measured by real-time quantitative polymerase chain reaction. Total RNA was extracted using a RiboPure-Blood Kit (ThermoFisher Scientific, AM 1928). RNA yield and purity were assessed using a BioSpec-nano spectrophotometer (Shimadzu, Tokyo, Japan), and integrity was determined following native agarose gel (2%) electrophoresis. cDNA was synthesized from high-quality RNA using an iScript cDNA synthesis kit (BioRad, Hercules, CA, USA). Primers (IL-8, MCP1, TNFα, IL6, TLR4, p65, β-actin) were purchased from Integrated DNA Technologies (Table S3) and validated from in-house melt curve and standard curve analysis. Analysis was performed using a Bio-Rad CFX384 instrument with SYBR Green Supermix (Bio-Rad). Target gene expression, with normalization to β-actin, was calculated using the 2^−ΔΔCT^ method [38].

### 2.8. Intestinal Permeability

Intestinal permeability was determined based on 24 h urinary excretion of lactulose, mannitol, sucralose, and erythritol, which were ingested as part of the test meal challenge. In brief, an aliquot of collected urine was diluted in ultrapure water, mixed with internal standards (^13^C_12_-lactulose (Sigma, St. Louis, MO, USA) ^13^C_6_-glucose (Sigma), and ^13^C_6_-mannitol (Cambridge Isotopes Laboratories, Tewksbury, MA, USA), and centrifuged (10,000× *g*, 6 min, 4 °C). The supernatant was collected, mixed with acetonitrile/water (85:15, *v*/*v*), and centrifuged again. The resulting supernatant was analyzed by LC-MS, as we previously described [39]. Upper and lower gastrointestinal permeability is based on urinary excretion ratios of lactulose/mannitol from 0–5 h and sucralose/erythritol from 5–24 h. Region-specific gastrointestinal permeability is based on lactulose being absorbed, paracellularly at the small intestine, and with normalization to mannitol that is absorbed transcellularly [40]. Because lactulose is hydrolyzed by bacteria present in the colon [41], colonic permeability is based on paracellular absorption of sucralose and with normalization to erythritol that is absorbed transcellularly, and consistent with neither sugar being affected by colonic bacteria [42].

### 2.9. Short-Chain Fatty Acids

A panel of 5 straight-chain SCFAs (acetate, propionate, butyrate, valerate, caproate) and 4 branched-chain SCFAs (isobutyrate, 2-methylbutyrate, isovalerate, 4-methylvalerate) were assessed by LC-MS, as we described previously [39]. In brief, fecal samples were homogenized in 1:1 acetonitrile/water using a Bead Mill 24 Homogenizer (Fisherbrand, Waltham, MA, USA) in tubes containing 1.0 mm zirconia/silica beads (ThermoFisher Scientific) and centrifuged (15,000× *g*, 10 min, 10 °C). The supernatant was mixed with internal standard (^13^C_4_-butyrate; Cambridge Isotopes Laboratories) and derivatized with 3-nitrophenylhydrazine to convert SCFAs to their corresponding 3-nitrophenylhydrazones prior to LC-MS analysis.

### 2.10. Statistical Analysis

The primary outcome of this study was serum endotoxin. Although no human studies have examined MFGM on endotoxemia, study powering was based on our data [3] that serum endotoxin in MetS persons is approximately doubled those of healthy persons (32.4 ± 4.4 vs. 16.4 ± 7.8 EU/mL) and our prediction that MEB would reduce fasting endotoxin by 25%. Our power analysis indicated that 14 subjects would be needed to reject the null hypothesis with 90% power (α = 0.05). However, to account for potential attrition and to consider a gender × treatment interaction, we enrolled 24 individuals with MetS.

Data (means ± SE) were analyzed using GraphPad Prism (version 9.5.0) or R software (4.1.2). Fisher’s exact test was used on categorical data of participant characteristics while an unpaired Student’s *t*-test was used on the nominal data. Initial analyses were performed using repeated measures three-way ANOVA to consider effects due to sex, time (day 0 vs. day 13), treatment (MEB vs. COMP), and their interactions. Because no sex differences were detected for the primary endpoint, repeated measures two-way ANOVA was used to evaluate main effects due to time, treatment, and their interaction. A Student’s *t*-test (paired) was used to assess between-treatment effects for SCFAs, urinary sugars, and the area under the curve during the postprandial period (AUC_0–180min_), which was calculated for each participant using the trapezoidal rule. Postprandial data are expressed as changes from fasting concentrations on day 13. Multiple linear regression, accounting for within-subject repeated measures, was used to evaluate correlations between study variables using the rmcorr package in R as described [43]. Statistical significance for all analyses was set at *p* ≤ 0.05.

## 3. Results

### 3.1. Participants

**Figure 1:**
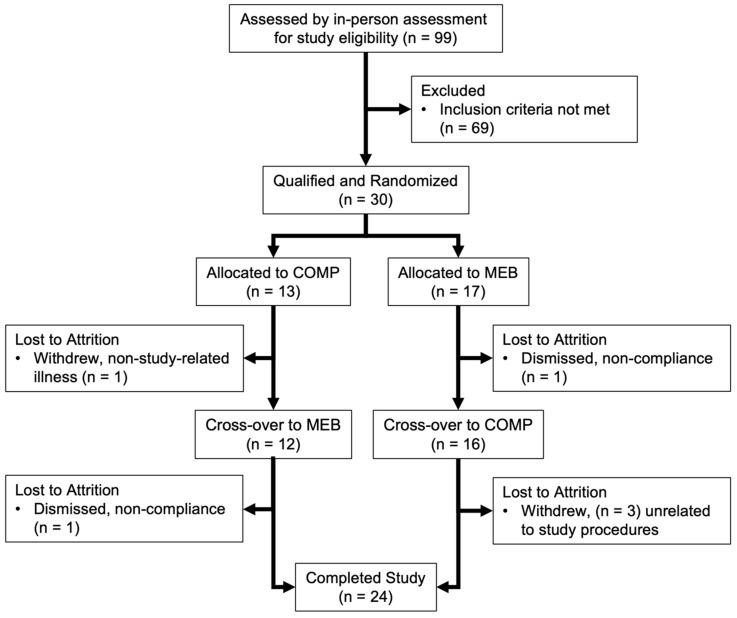
Flow of persons with metabolic syndrome through the randomized controlled trial examining two-week daily consumption of MEB or COMP. Participants completed the study with no adverse events, but 4 were lost to follow-up and 2 were dismissed due to non-compliance to study procedures. Abbreviations: COMP, comparator beverage; MEB, milk fat globule membrane-enriched beverage.

A total of 30 persons with MetS fulfilling study eligibility criteria were enrolled in the clinical trial (Figure 1). However, two participants were dismissed due to non-compliance to the prescribed diet; one participant was lost to attrition due to illness unrelated to the study; and three participants elected not to complete the alternate study arm once institutional research activities resumed during the global pandemic. Thus, 24 individuals with MetS completed all study aspects in agreement with our planned recruitment and were included in the final data analysis. No participants experienced any adverse effects related to study procedures. All participants (*n* = 11 men, 13 women) met the minimum three of five established criteria for MetS (Table 2), with 79% fulfilling three criteria and 21% fulfilling four criteria; none fulfilled five criteria. Most participants had increased waist circumference (88%) and depressed HDL-C (96%). Elevated blood glucose was present in 71% of participants, followed by high blood pressure (42%) and hypertriglyceridemia (25%). No sex differences were observed in any MetS criteria (*p* > 0.05).

### 3.2. Test Beverage and Diet Compliance

**Figure 2:**
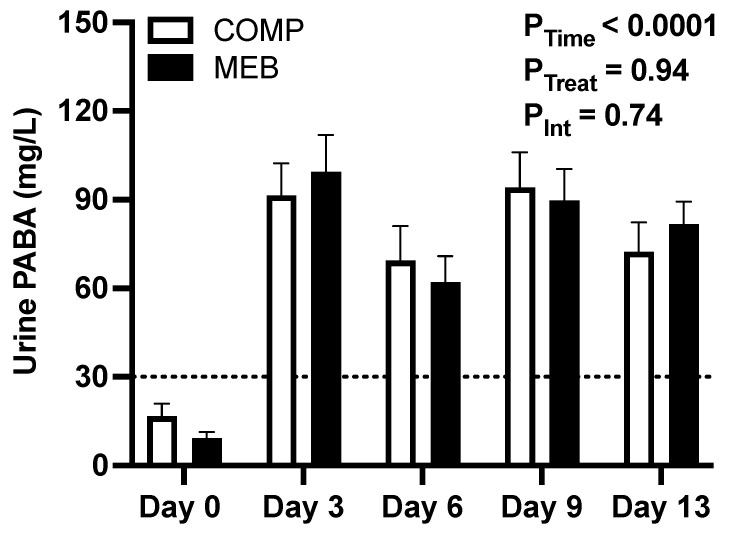
Compliance to test beverage consumption in persons with metabolic syndrome who consumed three daily servings (250 mL) of MEB or COMP for 2 weeks. PABA was added to all test beverages, and urinary PABA was assessed 5 times during each study arm from spot urine samples. Urinary PABA at >30 mg/L was the threshold for compliance based on a prior report [29]. Data (means ± SE, *n* = 24) were analyzed by two-way repeated measures ANOVA. *p* ≤ 0.05 was considered statistically significant. Abbreviations: COMP, comparator beverage; MEB, milk-fat-globule-membrane-enriched beverage; PABA, para-aminobenzoic acid.

Compliance to test beverages was 95% based on returned bottles. Urinary PABA was also measured to corroborate compliance (Figure 2). On average, day 3–13 spot urine PABA concentrations increased well above (*p* < 0.0001) the pre-established compliance threshold of >30 mg/dL [29] and without any between-treatment difference (*p* = 0.94) or treatment x time interaction (*p* = 0.74). Inspection of participants’ urinary PABA values also indicated that 82–86% of all spot urines from both treatment arms were >30 mg/L.

Consistent with our prescribed diet approach, participants’ energy and macronutrient intakes did not differ between trials (*p* > 0.05; Table 3). On average, daily energy intakes during each two-week intervention were 2178 ± 77 kcal in COMP and 2212 ± 70 kcal in MEB compared with prescribed energy intakes (2450 ± 64 kcal). These data indicate that participants consumed ≈90% of their prescribed energy intakes, consistent with our approach to provide a prescribed diet that marginally exceeded calculated energy requirements. Further, only 2.4–3.2% of daily energy was from non-prescribed foods, regardless of study arm, with no between-trial difference observed (*p* > 0.05). Overall, in addition to no between-trial differences in energy, participants’ intakes of carbohydrates, fiber, protein, and fat did not differ between trials. Lastly, neither total nor subcategory HEI-2015 scores differed between treatment arms (*p* > 0.05; Table 3). Overall, findings of urinary PABA and dietary assessment support strong compliance to the intervention examining the potential benefits of MEB on cardiometabolic health.

### 3.3. Anthropometrics, Blood Pressure, and Fasting Cardiometabolic Markers

Participants’ waist circumference, systolic blood pressure, or diastolic blood pressure were not affected by time, treatment, or their interaction (Table 4). However, BMI decreased from day 0 to day 13 by ≈0.3 kg/m^2^, regardless of treatment (*p* < 0.01), due to a 0.75 kg loss in body mass.

Fasting glucose, insulin, triglyceride, GLP-1, and calculated insulin resistance (HOMA-IR) did not change significantly within or between treatments (Table 4). GIP was lower during MEB, but this occurred without any time x treatment interaction. Plasma HDL-C concentration showed a small but statistically significant decrease, regardless of treatment arm. Total cholesterol and LDL-C showed a significant time x treatment interaction, such that they were unaffected during MEB but decreased during COMP to concentrations lower than those on day 13 in MEB.

Fasting endotoxin, the primary outcome of this randomized controlled trial, did not differ on day 0 between study treatments (Table 4). Contrary to our hypothesis, its circulating concentrations during the intervention were unaffected by MEB nor were there any time or treatment x time effects. We also considered LBP:sCD14 as an alternative biomarker of endotoxin exposure [44]. While a within-trial decrease in circulating CD14 was observed regardless of test beverage, and LBP concentrations were higher during MEB regardless of time, there were no time, treatment, or time x treatment effects observed for LBP:sCD14. Thus, MEB did not improve metabolic endotoxemia in persons with MetS.

### 3.4. Postprandial Excursions in Cardiometabolic Markers

**Figure 3:**
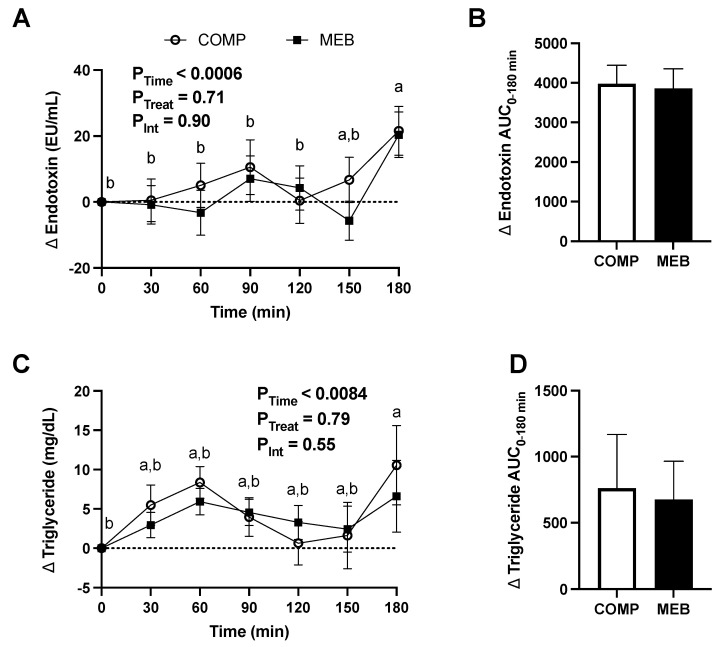
Postprandial serum endotoxin and plasma triglyceride in persons with metabolic syndrome who consumed MEB or COMP for 2 weeks prior to ingesting a high-fat/high-carbohydrate test meal challenge. Data (means ± SE, *n* = 24) are expressed as change from baseline (fasting) concentrations during the three-hour postprandial period. AUC_0–180min_ was calculated using the trapezoidal rule. Two-way repeated measures ANOVA was used to evaluate effects due to time, treatment, and their interaction during the postprandial period. Means not sharing a common letter are significantly different from each other based on post hoc analysis following a main effect due to time. A paired Student’s *t*-test was used to evaluate between-treatment effects on AUC_0–180min_. *p* ≤ 0.05 was considered statistically significant. (**A**) Serum endotoxin was unaffected by MEB but increased over time, regardless of treatment. (**B**) Endotoxin AUC_0–180min_ was not different between treatments. (**C**) Plasma triglyceride increased postprandially, regardless of test beverage, and was unaffected by MEB. (**D**) Triglyceride AUC_0–180min_ did not differ between MEB and COMP. Abbreviations: AUC, area under the concentration curve; COMP, comparator beverage; MEB, milk-fat-globule-membrane-enriched beverage.

Prior reports show that a high-fat meal challenge significantly increases circulating endotoxin [45] and that endotoxemia closely associates with postprandial hypertriglyceridemia [46]. In agreement, ingestion of a high fat/high carbohydrate meal challenge in the present study significantly increased serum endotoxin (Figure 3A,B) and plasma triglyceride (Figure 3C,D), regardless of test beverage treatment. However, the AUC_0–180min_ of endotoxin and triglyceride showed no difference between treatments (*p* > 0.05). Endotoxin AUC and triglyceride AUC also were not correlated with each other (*p* = 0.54).

**Figure 4:**
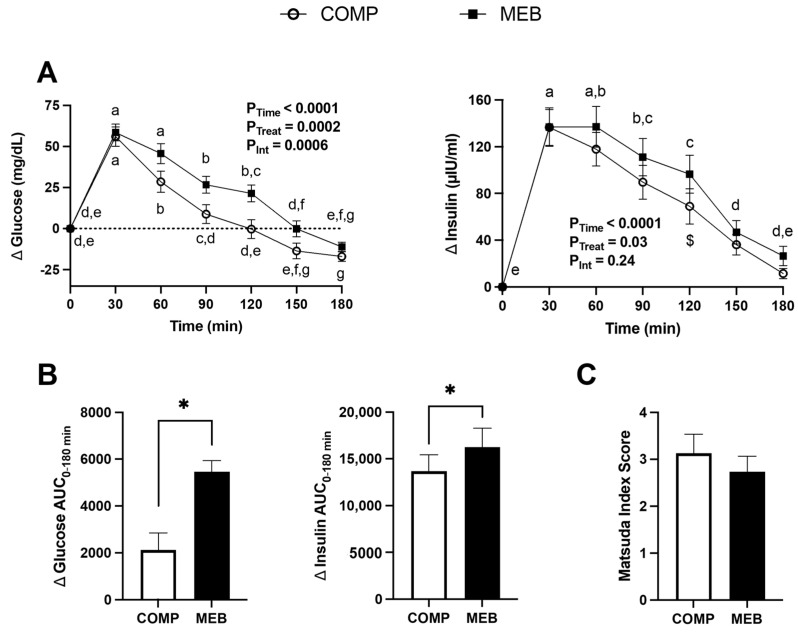
Postprandial plasma glucose, insulin, and Matsuda index in persons with metabolic syndrome who consumed MEB or COMP for 2 weeks prior to ingesting a high-fat/high-carbohydrate test meal challenge. Data (means ± SE, *n* = 24) are expressed as change from baseline (fasting) concentrations during the three-hour postprandial period. AUC_0–180min_ was calculated using the trapezoidal rule. Two-way repeated measures ANOVA was used to evaluate effects due to time, treatment, and their interaction during the postprandial period. Means not sharing a common letter are significantly different from each other based on post hoc analysis following a main effect due to time. A main effect due to treatment was also detected, and $ indicates a significant difference following post hoc analysis. A paired Student’s *t*-test was used to evaluate between-treatment effects on AUC_0–180min_ (*, *p* < 0.05). *p* ≤ 0.05 was considered statistically significant. (**A**) Plasma glucose and insulin increased over time but were unaffected by MEB. (**B**) Glucose and insulin AUC_0–180min_ were higher in MEB compared to COMP. (**C**) Matsuda Index Score did not differ between MEB and COMP. Abbreviations: AUC, area under the concentration curve; COMP, comparator beverage; MEB, milk-fat-globule-membrane-enriched beverage.

Because hyperglycemia also promotes the systemic influx of microbial products from the gut [8], we examined postprandial excursions of plasma glucose and insulin in response to the test meal challenge and their relationship to serum endotoxin (Figure 4). As expected, plasma glucose and insulin increased in response to the test meal challenge. However, a time × treatment interaction indicated that glucose concentrations similarly peaked at 30 min, regardless of test beverage treatment, but lowered over time more slowly in MEB compared with COMP (Figure 4A). Accordingly, plasma glucose AUC_0–180min_ was greater in MEB compared with COMP (Figure 4B). Although postprandial concentrations of insulin showed no time x treatment interactive effect (*p* = 0.24), a significant treatment effect indicated that plasma insulin was higher in MEB than COMP. Likewise, insulin AUC_0–180min_ was greater in MEB compared with COMP. However, after calculating the Matsuda index, an insulin resistance metric based on postprandial concentrations of glucose and insulin (18), no significant difference between test beverages was observed (Figure 4C). Endotoxin AUC also was not correlated with glucose AUC (*p* = 0.99).

**Figure 5:**
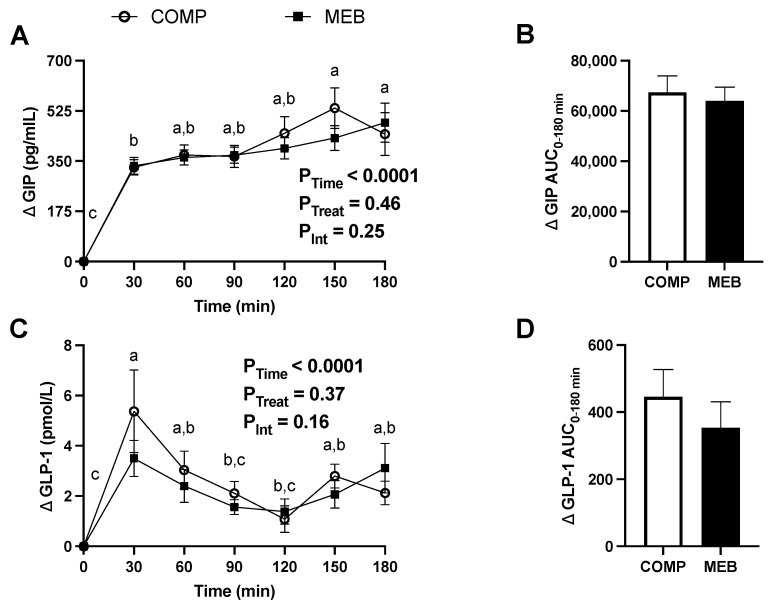
Postprandial plasma GIP and GLP-1 in persons with metabolic syndrome who consumed MEB or COMP for 2 weeks prior to ingesting a high-fat/high-carbohydrate test meal challenge. Data (means ± SE, *n* = 24) are expressed as change from baseline (fasting) concentrations during the three-hour postprandial period. AUC_0–180min_ was calculated using the trapezoidal rule. Two-way repeated measures ANOVA was used to evaluate time, treatment, and interactive effects during the postprandial period. Means not sharing a common letter are significantly different from each other based on post hoc analysis following a main effect due to time. A paired Student’s *t*-test was used to evaluate between-treatment effects on AUC_0–180min_. *p* ≤ 0.05 was considered statistically significant. (**A**) Plasma GIP was unaffected by MEB but increased over time, regardless of treatment. (**B**) GIP AUC_0–180min_ did not differ between treatments. (**C**) Plasma GLP-1 increased postprandially, regardless of test beverage, and was unaffected by MEB. (**D**) GLP-1 AUC_0–180min_ did not differ between MEB and COMP. Abbreviations: AUC, area under the concentration curve; COMP, comparator beverage; GIP, glucose-dependent insulinotropic polypeptide; GLP-1, glucagon-like peptide; MEB, milk-fat-globule-membrane-enriched beverage.

Lastly, postprandial concentrations of GIP and GLP-1 only showed a main effect of time, regardless of test beverage treatment (*p* < 0.0001; Figure 5), and there was no between-treatment difference in the GIP AUC_0–180min_ or GLP-1 AUC_0–180min_ (Figure 5). Overall, these findings indicate that MEB does not improve metabolic endotoxemia nor fasting or postprandial metabolic excursions in persons with MetS.

### 3.5. Gut Barrier Function and Inflammation

**Figure 6:**
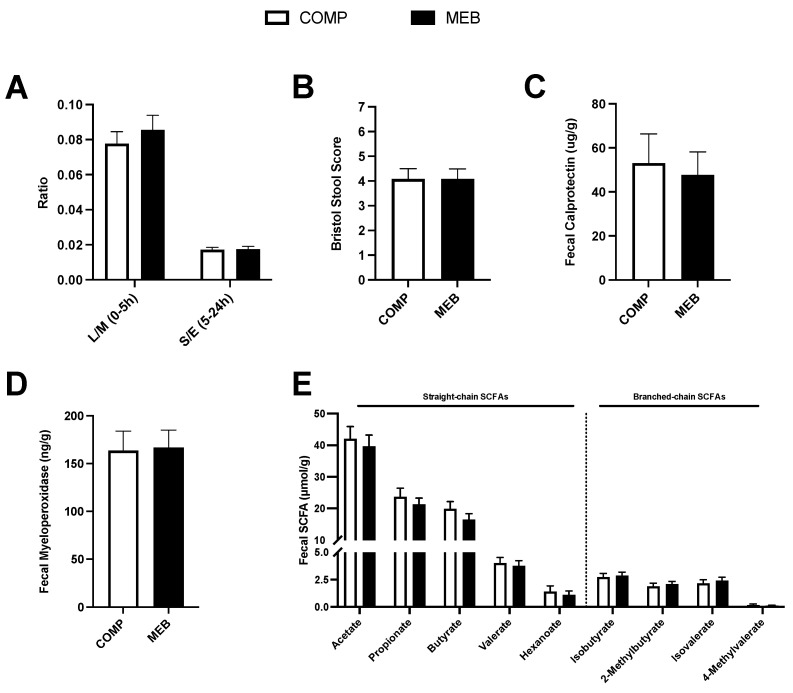
Gut permeability, inflammation, and SCFAs in persons with metabolic syndrome who consumed MEB or COMP for 2 weeks prior to ingesting a high-fat/high-carbohydrate test meal challenge. Data (means ± SE, *n* = 24) were analyzed by a paired Student’s *t*-test. *p* < 0.05 was considered statistically significant. (**A**) Urinary 0–5 h L/M and 5–24 h S/E did not differ between treatments. (**B**) Bristol stool scores, (**C**) fecal myeloperoxidase, and (**D**) fecal calprotectin did not differ between MEB and COMP. (**E**) Fecal straight-chain and branched-chain SCFAs did not differ between treatments. Abbreviations: COMP, comparator beverage; L/M, lactulose/mannitol; MEB, milk-fat-globule-membrane-enriched beverage; SCFA, short-chain fatty acids; S/E, sucralose/erythritol.

Participants completed a gastrointestinal permeability test at the end of each study arm to test the hypothesis that MEB would alleviate ‘leaky gut’ in association with lowered intestinal inflammation. Contrary to our hypothesis, small intestinal permeability based on urinary lactulose/mannitol (0–5 h) and colonic permeability based on urinary sucralose/erythritol (5–24 h) were not significantly different between MEB and COMP (*p* = 0.45–0.79; Figure 6A). We also observed no difference between treatments in stool characteristics (Figure 6B), nor were fecal concentrations of calprotectin or myeloperoxidase different between MEB and COMP (Figure 6C,D).

We also considered that fecal SCFAs, which regulate gut barrier health and inflammation [47], would be improved by MEB. However, concentrations of total SCFAs nor total straight-chain or total branched-chained SCFAs (with or without normalization to total SCFAs) were not different between study treatments. There were also no between-treatment differences in concentrations of five straight-chain SCFAs or four branched-chain SCFAs (Figure 6E).

### 3.6. Systemic Inflammation

**Figure 7:**
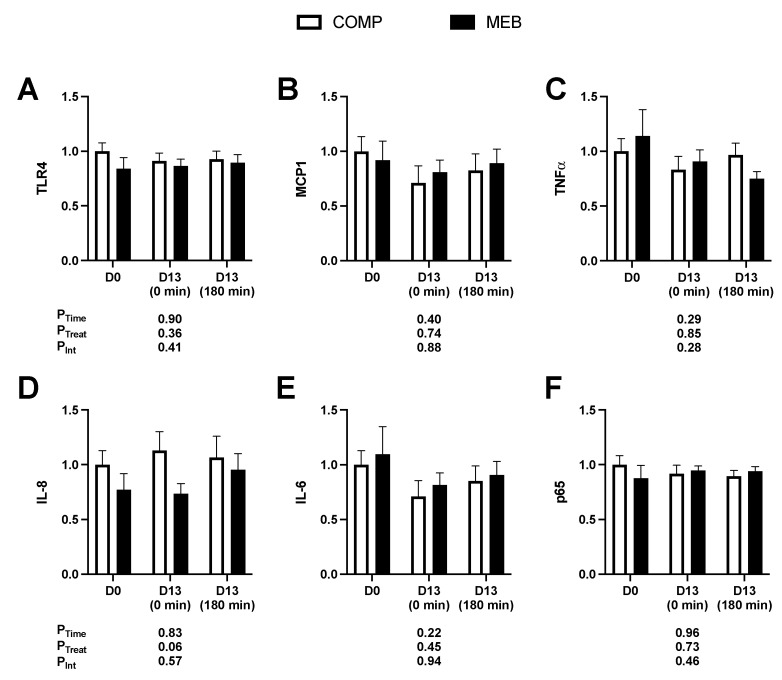
Whole blood expression of inflammatory genes in persons with metabolic syndrome who consumed MEB or COMP for 2 weeks prior to ingesting a high-fat/high-carbohydrate test meal challenge. Data (means ± SE, *n* = 24) were normalized to β-actin and analyzed by two-way RM ANOVA. (**A**) TLR4, (**B**) MCP1, (**C**) TNFα, (**D**) IL-8, (**E**) IL-6, and (**F**) p65 showed no effects due to time, treatment, or their interaction (*p* > 0.05 for all). Abbreviations: COMP, comparator beverage; IL, interleukin; MEB, milk-fat-globule-membrane-enriched beverage; MCP1, monocyte chemoattractant protein; TLR4, Toll-like receptor-4; TNFα, tumor necrosis factor-α.

RNA was isolated from fasting whole blood on day 0 and on day 13 prior to and at 180 min post-test meal challenge. Consistent with gut inflammation being unaffected (Figure 6C,D), mRNA expression of six genes involved in TLR4/NFκB inflammation (Figure 7A–F) did not differ between trials at baseline (day 0), at fasting after each intervention period (day 13), or in response to the meal challenge on day 13. There were also no main effects of time or time x treatment interactions for mRNA expression for fasting levels (day 0 vs. day 13) or postprandial levels (day 13, 0 vs. 180 min). We did, however, observe that endotoxin AUC_0–180min_ significantly correlated with day 13 (0 min) expression levels of TLR4 (r_rm_ = 0.70; *p* = 0.003), and fasting serum endotoxin at day 13 was correlated with p65 (r_rm_ = 0.57; *p* = 0.03), supporting the fact that endotoxemia contributes to host inflammation.

## 4. Discussion

This rigorously controlled, randomized crossover trial demonstrated, contrary to our hypothesis, that two-week consumption of an MFGM-enriched full-fat milk beverage does not affect intestinal-level or circulating biomarkers of cardiometabolic health in MetS persons. Metabolic endotoxemia, based on complementary assessments of serum endotoxin and LBP:sCD14 at fasting or postprandially following a meal challenge, was unaffected by MEB compared with COMP. Corroborating findings also show that MEB did not affect expression of TLR4/NFκB inflammatory responses. MEB also had no effect on insulin resistance (HOMA-IR) or insulin sensitivity (Matsuda index), nor did it influence gut incretins. Lastly, despite compelling evidence from rodent models [17,48], intestinal permeability, intestinal inflammation, and fecal SCFAs were unaffected by the MFGM-enriched milk. Overall, this RCT that was designed for research translation of preclinical evidence [21] did not demonstrate cardiometabolic benefits of MFGM-enriched dairy milk. However, neutral outcomes of this short-term RCT provide contradictory evidence against a long-standing controversy, based largely on observational evidence, suggesting that full-fat dairy milk adversely affects cardiometabolic health [15].

Whether dairy foods, regardless of their fat content, impact cardiometabolic health has been long debated [49]. Despite limited evidence from RCTs that can demonstrate causality, leading health authorities recommend 2–3 daily servings of low-fat or non-fat varieties over full-fat dairy milk to support human health [33]. This recommendation is derived largely from early observational studies under the premise that higher energy and saturated fat intakes, such as those from full-fat dairy milk, increase the risk of cardiometabolic disorders [50]. However, evaluating the benefits of dairy foods presents challenges [51,52], especially in observational studies, because significant heterogeneity exists in the number and composition of dairy foods; dietary exposures are difficult to accurately capture due to limited dietary assessment tools and food recall bias; and potential exists for co-linearity when dairy foods are evaluated from complex dietary patterns. Thus, RCTs that directly evaluate full-fat dairy foods on cardiometabolic health are needed to resolve equivocal evidence. For example, a meta-analysis of RCTs indicated that dairy intakes, without regard of their fat content, have no effect on body weight in studies without energy restriction but some benefit on body fat when dietary energy is restricted [53]. Others report in a meta-analysis of cohort studies that total dairy intakes had no benefit on body weight, whereas yogurt was inversely associated with obesity risk [54]. Well-controlled RCTs that specifically consider dairy fat content are also important because the evidence supports health benefits, including satiety and glycemic control [55]. Further, full-fat milk contains bioactive constituents (e.g., polar lipids) as part of the MFGM [15], which help to manage dyslipidemia and inflammation [48]. Thus, our RCT was designed to evaluate cardiometabolic benefits of full-fat dairy milk, with emphasis on MFGM. This provided rationale to formulate MEB identical to commercial full-fat milk except for its enrichment with MFGM. We then compared it to a milk-like beverage having identical dairy proteins and carbohydrate, a similar fatty acid profile but derived from plant oils, and equivalent total polar lipid content derived from soy lecithin but differing significantly in composition from those in MFGM.

The present RCT had rigorous dietary control to limit heterogeneity of the basal diet within and between participants and to promote weight stability to evaluate the independent effects of MEB. High compliance to the prescribed diet was evidenced based on no between-trial differences in energy or macronutrients, that 97–98% of total energy intakes were from administered foods, and that between-trial dietary patterns did not differ based on HEI-2015 scores. While a small but significant 0.75 kg decrease in body mass occurred in both study arms, this is unlikely to be confounding consistent with the lack of within-trial (time)-dependent effects on blood pressure and fasting concentrations of glucose, insulin, triglyceride, and total cholesterol. Scientific rigor was also enhanced by complementary assessments of compliance to test beverage consumption. We achieved 95% compliance based on returned beverage bottles and objectively confirmed ingestion by measures of spot urine PABA, which was added to all test beverages. On average, urinary PABA increased substantially, and 82–86% of all spot urines met the pre-established concentration threshold to achieve compliance. The small discrepancy between bottle counts and spot urines may reflect inflated compliance when bottle counts are used or could reflect a delay between when the test beverage was consumed relative to when the spot urine was collected; PABA has a rapid rate of elimination [30]. Regardless, study compliance was confirmed to be high thereby permitting rigorous evaluation of the hypothesis that MEB would alleviate metabolic endotoxemia.

The primary outcome of this RCT was an expected decrease in serum endotoxin in response to MEB. We focused on endotoxin because metabolic endotoxemia is implicated as a cause of obesity and insulin resistance [4], it occurs in persons with MetS [3], and an MFGM-derived polar lipid (sphingomyelin) tended to decrease endotoxemia in obese mice fed a high-fat diet [17]. Further, mice fed a diet formulated with milk fat containing 10% MFGM protected against LPS-induced intestinal permeability and premature mortality in association with lower circulating pro-inflammatory cytokines (i.e., TNFα, IL-6, MCP-1, IFN-γ) compared with mice fed a diet formulated with corn oil [19]. Contrary to our hypothesis, MEB did not attenuate serum endotoxin at fasting or postprandially following a test meal challenge. It also did not decrease the ratio of LBP:sCD14, a biomarker of endotoxin exposure that is demonstrated to decrease following chronic yogurt consumption in non-obese and obese women [56]. The lack of MEB-mediated improvement in metabolic endotoxemia was unexpected and is corroborated by several mechanistic endpoints that were evaluated in this RCT.

First, because metabolic endotoxemia results from enhanced translocation of gut-derived endotoxins to the systemic circulation, we considered that MFGM-enriched MEB would decrease intestinal permeability. Indeed, MFGM supplementation promotes development of the intestinal epithelium and improves tight junction protein patterns, at least in neonatal rat pups [57]. MFGM also decreased cellular permeability and upregulated the expression of the tight junctions claudin and zonula occluden in a Caco-2 human enterocyte model [58]. However, we showed that neither small intestine nor colonic permeability are improved by MEB compared with COMP. Further, there was a lack of intestinal-level anti-inflammatory effect on measures of fecal calprotectin and myeloperoxidase by MEB, suggesting that neutrophil infiltration and oxidative injury to the intestinal epithelium were unaffected.

Second, we considered that the reported benefits of MFGM and/or full-fat dairy milk on glycemic control [59] and dyslipidemia [17] would help to limit the absorption of gut-derived endotoxins. This is consistent with hyperglycemia driving intestinal permeability by GLUT2-dependent transcriptional reprogramming of epithelial cells and impairing tight junction integrity [8]. However, fasting glucose was not improved in our MetS participants in response to a controlled diet that included three daily servings of MEB. In addition, there was a lack of metabolic adaptation to MEB to attenuate postprandial glycemia following a high-fat/high-carbohydrate test meal challenge. On the basis of separate measures of glucose and insulin AUC, our data could suggest greater glucose intolerance during MEB compared to COMP. However, this is not corroborated when participants’ insulin sensitivity is calculated by the Matsuda index. Indeed, the Matsuda index estimates insulin sensitivity based on a composite formula that considers postprandial excursions in both glucose and insulin. Further, Matsuda scores but not AUCs of glucose or insulin are well-correlated with insulin sensitivity when determined via ‘gold standard’ euglycemic clamp studies [36]. Thus, we base our conclusion that MEB did not affect insulin sensitivity compared with COMP on the more comprehensive metric of Matsuda index, but also recognize that glucose tolerance based on glucose AUC is widely used to diagnosis diabetes. Therefore, the evidence indicates that MEB did not protect against hyperglycemia-induced increases in paracellular intestinal permeability that is known to lead to systemic influx of gut-derived endotoxin [8]. These findings are also in agreement with our observation that gut-health-promoting fecal SCFAs (e.g., butyrate) that upregulate tight junction expression [47], provide anti-inflammatory activity [47], and stimulate incretin secretion [60] were unaffected by MEB, despite evidence supporting MFGM to improve microbiota composition and function [17,20]. Alternatively, endotoxin can be transcellularly absorbed and packaged into chylomicrons following the same pathway as dietary lipid [46]. However, in the present study, circulating endotoxin increased in response to the high-fat/high-carbohydrate test meal challenge and without any between-treatment differences. This suggests that MEB, which was highly enriched in polar lipid that is known to limit absorption of dietary lipid (i.e., cholesterol) [61], did not protect against postprandial endotoxemia.

The present study was designed, in part, to establish efficacy and mechanistic insight of MEB to protect against MetS-associated complications, consistent with epidemiological studies indicating that higher intakes of full-fat dairy milk protect against cardiometabolic disorders [62]. Indeed, a large, multi-country prospective study (*n* = 112,992) suggested that full-fat dairy milk was associated with a lower MetS prevalence [14]. Mozaffarian et al. [63] reported higher circulating concentrations of odd-chain fatty acids (i.e., 15:0, 17:0, t − 16:1*n* − 7), which are found in full-fat dairy milk, were associated with a 46% lower risk of incident type 2 diabetes. Others also report in a prospective observational study (*n* = 18,438, 11.2 y follow-up) that higher consumption of full-fat dairy products, but not low-fat dairy, was associated with less weight gain in adult women [64]. Despite these foundational observations, our RCT demonstrates that full-fat dairy milk, provided as MFGM-enriched MEB, does not adversely affect or improve cardiometabolic risk, consistent with findings of several observational studies [65].

Although our RCT was supported by strong rationale [21] and rigorously controlled, several aspects may have contributed to the lack of observed cardiometabolic benefit of MEB. We carefully considered milk dose (three servings/d) in agreement with observational evidence supporting benefits of full-fat dairy milk [14] and specifically enriched it with MFGM at levels ≈10 times that typically found in fluid milk [27]. Thus, the dose was appropriate, but the tw-week exposure period may have been insufficient for physiological adaptations to occur. Alternatively, benefits of MEB or its bioactive constituents may only be observed when directly co-ingested with an obesogenic diet or atherogenic dietary constituents. For example, mice fed a high-fat diet supplemented with milk sphingomyelin are protected from liver steatosis and inflammation [66]. Others report that the concurrent ingestion of high-dose milk polar lipids limits cholesterol absorption in humans [61] and MFGM as part of a high-fat meal protects against postprandial increases in cholesterol, insulinemia, and select inflammatory markers in overweight and obese individuals, especially those with elevated C-reactive protein [59]. However, we specifically did not administer test beverages with the high-fat-/high-carbohydrate challenge to recapitulate epidemiological studies that capture complete dietary patterns rather than meal-specific food intakes. Lastly, unlike cellular studies [18] and rodent studies [48] that have demonstrated health benefits of MFGM or its polar lipids at pharmacological levels, our dose of MFGM within full-fat milk was high but within intakes that can be readily achieved from a diet rich in dairy products beyond fluid milk. Thus, the present study supports research translation of epidemiological studies.

## 5. Conclusions

Overall, findings of this acute RCT show that MFGM-enriched MEB does not alleviate metabolic endotoxemia by improving gut barrier function or glycemia, suggesting that it does not adversely impact cardiometabolic health compared with an alternative milk-like beverage formulated with plant phospholipid and oils. While this short-term study suggests that MEB neither improves nor impairs cardiometabolic health, longer-term interventions are needed to better recapitulate outcomes from observational studies that generally consider longer time frames of exposure to dairy milk. Overall, these results provide evidence in contradiction of public health messages that have long-associated full-fat dairy milk with enhanced cardiometabolic risk. Indeed, our data show that dietary inclusion of MEB (or COMP) can help facilitate a small, but statistically significant, decrease in body mass that warrants detailed assessment in long-term intervention. Further, while MEB and COMP had identical carbohydrate and protein ingredients, as well as similar fatty acid profiles, their major difference was the sphingomyelin content. Sphingomyelin has demonstrated prebiotic and anti-microbial activities in vitro and in rodents [67], but longer-term study is required to establish whether its benefits on microbiota composition and function alleviate cardiometabolic risk and/or predict inter-individual responses to its ingestion. Future studies that integrate multi-omics workflows may therefore help to establish mechanisms by which full-fat dairy milk and/or its bioactive MFGM fraction protect against cardiometabolic disorders. While such studies will help to reconcile the equivocal observational evidence surrounding the health benefits of dairy milk, the present study suggests that, at worst, full-fat dairy milk has neutral influence on cardiometabolic risk.

## Figures and Tables

**Table 1:** Composition of test beverages used in the randomized controlled trial ^1^.

Component	COMP (250 mL)	MEB (250 mL)
Energy (kcal)	170	162
Total carbohydrates (g)	14.8	13.2
Total protein (g)	8.4	8.4
Total fat (g)	7.4	7.1
Saturated (g)	5.3	4.4
Monounsaturated (g)	1.8	2.1
Polyunsaturated (g)	0.3	0.6
Trans (mg)	0.0	4.6
Total phospholipids (mg)	770.0	772.5
Sphingomyelin (mg)	0.0	125.9
Phosphatidylserine (mg)	0.0	108.7
Phosphatidylcholine (mg)	185.2	171.7
Phosphatidylethanolamine (mg)	206.9	260.2
Phosphatidylinositol (mg)	175.4	63.7
Other phospholipids (mg) ^2^	202.3	42.3
C4:0 (mg)	0.0	205.5
C6:0 (mg)	0.0	143.7
C8:0 (mg)	112.7	99.2
C10:0 (mg)	112.0	216.3
C12:0 (mg)	939.8	261.0
C14:0 (mg)	490.8	779.6
C16:0 (mg)	2537.4	2048.6
C18:0 (mg)	402.6	800.4
C20:0 (mg)	2.5	3.2
C14:1 (mg)	0.00	26.1
C16:1 (mg)	0.72	115.2
C18:1 (mg)	2186.7	1596.0
C18:2 (mg)	650.0	223.2
C18:3 (mg)	3.6	35.8

^1^ COMP was formulated with soy phospholipids and a palm oil/coconut oil blend (75:25), and MEB was formulated with MFGM and butter oil. ^2^ Unidentified phospholipids. Abbreviations: COMP, comparator beverage; MEB, milk fat globule enriched beverage, MFGM, milk fat globule membrane.

**Table 2:** Baseline characteristics of MetS participants at screening ^1^.

	All (*n* = 24)	Men (*n* = 11)	Women (*n* = 13)	*p*
Age (y)	37.3 ± 2.1	33.6 ± 2.1	40.4 ± 3.2	0.11
BMI (kg/m^2^)	33.4 ± 1.1	32.9 ± 1.4	33.8 ± 1.7	0.70
Waist circumference (cm)	106.6 ± 2.1	108.2 ± 3.2	105.2 ± 2.9	0.50
SBP (mmHg)	120.4 ± 2.3	124.6 ± 2.7	116.9 ± 3.4	0.09
DBP (mmHg)	83.1 ± 2.1	87.0 ± 2.7	79.9 ± 3.0	0.10
Plasma triglyceride (mg/dL)	130.1 ± 16.0	149.8 ± 30.3	113.5 ± 14.4	0.27
Plasma HDL-C (mg/dL)	37.2 ± 1.7	34.2 ± 2.7	39.7 ± 2.1	0.12
Plasma glucose (mg/dL)	101.8 ± 1.8	103.7 ± 2.8	100.2 ± 2.4	0.34
MetS criteria				
Three risk factors (%)	79	64	92	0.14
Four risk factors (%)	21	36	8	0.14
Five risk factors (%)	0	0	0	-
Waist circumference (%)	88	73	100	0.08
HDL-C (%)	96	91	100	0.46
Glucose (%)	71	73	69	0.99
Blood pressure (%)	42	64	23	0.09
Triglyceride (%)	25	36	15	0.36

^1^ Values are means ± SE or the proportion of participants meeting specific MetS-related criteria. Participants were classified with MetS based on meeting ≥3 of the 5 established MetS criteria [22]. Between-sex comparisons were performed using Fisher’s exact test for categorical data and unpaired Student’s *t*-test for nominal data. Abbreviations: BMI, body mass index; DBP, diastolic blood pressure; HDL-C, high-density lipoprotein-cholesterol; MetS, metabolic syndrome; SBP, systolic blood pressure.

**Table 3:** Dietary intakes of persons with metabolic syndrome who completed the crossover randomized control trial examining daily consumption of MEB and COMP ^1^.

	COMP	MEB	*p* ^1^
Prescribed energy (kcal) ^2^	2450 ± 64	2450 ± 64	-
Consumed energy (kcal) ^2^	2178 ± 77	2212 ± 70	0.06
Carbohydrate (% kcal)	54.2 ± 0.31	54.3 ± 0.27	0.28
Fat (% kcal)	29.4 ± 0.19	29.8 ± 0.17	0.70
Protein (% kcal)	18.6 ± 0.17	18.4 ± 0.14	0.19
Non-prescribed foods (% kcal)	2.4 ± 0.006	3.2 ± 0.01	0.47
Fiber (g)	16.4 ± 0.29	16.9 ± 0.26	0.11
HEI-2015 total score ^3^	62.2 ± 0.35	62.1 ± 0.36	0.83
HEI-2015 adequacy components			
Total fruits	4.6 ± 0.06	4.6 ± 0.06	0.73
Whole fruits	4.6 ± 0.06	4.7 ± 0.06	0.41
Total vegetables	3.7 ± 0.01	3.7 ± 0	0.06
Greens and beans	0.3 ± 0.02	0.3 ± 0.02	0.66
Whole grains	1.6 ± 0.07	1.6 ± 0.07	0.33
Dairy	0	0	-
Total protein foods	4.4 ± 0.06	4.5 ± 0.05	0.67
Seafood and plant proteins	2.3 ± 0.13	2.2 ± 0.13	0.85
Healthy fats	9.8 ± 0.09	9.8 ± 0.09	0.47
HEI-2015 moderation components			
Refined grains	3.9 ± 0.16	3.6 ± 0.16	0.21
Sodium	10 ± 0.02	9.8 ± 0.07	0.06
Added sugars	9.6 ± 0.05	9.6 ± 0.04	0.73
Saturated fat	9.9 ± 0.09	9.8 ± 0.08	0.47

^1^ Data (means ± SE, *n* = 24) were analyzed using a paired Student’s *t*-test. ^2^ Prescribed energy was determined based on the Harris–Benedict equation [31] with adjustment for physical activity. Consumed energy consumed was calculated from weighed food records during each 14-day intervention period. ^3^ Healthy Eating Index (HEI-2015) total score was calculated from the basal diet without inclusion of either test beverage.

**Table 4:** Clinical and biochemical endpoints before and after each two-week treatment with COMP or MEB in persons with metabolic syndrome ^1^.

	COMP		MEB				
Parameter	Day 0	Day 13	Day 0	Day 13	P_Time_	P_Treatment_	P_Interaction_
BMI (kg/m^2^)	33.4 ± 1.1	33.2 ± 1.1	33.7 ± 1.1	33.4 ± 1.1	0.0013	0.05	0.80
Waist circumference (cm)	107.5 ± 2.2	107.3 ± 2.2	108.0 ± 2.3	107.5 ± 2.5	0.25	0.81	0.87
SBP (mmHg)	119.9 ± 2.8	118.3 ± 2.0	121.4 ± 3.0	119.6 ± 2.6	0.64	0.65	0.76
DBP (mmHg)	81.8 ± 1.7	81.8 ± 1.7	84.9 ± 2.2	83.2 ± 2.1	0.73	0.07	0.45
Glucose (mg/dL)	101.2 ± 1.8	99.6 ± 2.5	101.6 ± 1.7	98.1 ± 1.6	0.10	0.71	0.42
Insulin (μIU/mL)	17.2 ± 9.9	14.5 ± 6.0	16.4 ± 6.5	15.1 ± 6.5	0.10	0.96	0.41
HOMA-IR	4.2 ± 0.4	3.5 ± 0.3	4.0 ± 0.3	3.6 ± 0.3	0.10	0.86	0.59
GIP (pg/mL)	101.9 ± 13.6 ^a^	90.6 ± 13.2 ^a^	87.3 ± 15.5 ^b^	67.0 ± 6.9 ^b^	0.12	0.006	0.63
GLP-1 (pmol/L)	7.4 ± 2.4	6.7 ± 2.2	6.1 ± 1.6	6.0 ± 1.6	0.05	0.24	0.13
Triglyceride (mg/dL)	109.2 ± 8.9	103.3 ± 9.9	106.0 ± 9.8	101.7 ± 8.0	0.49	0.61	0.83
Total cholesterol (mg/dL)	183.9 ± 8.9 ^a^	161.8 ± 8.3 ^b^	186.5 ± 9.8 ^a^	187.0 ± 8.9 ^a^	0.09	0.0019	0.0064
HDL-cholesterol (mg/dL)	36.2 ± 1.8 ^a^	34.1 ± 1.4 ^b^	36.5 ± 1.7 ^a^	32.3 ± 1.3 ^b^	0.0004	0.23	0.07
LDL-cholesterol (mg/dL) ^2^	125.8 ± 7.8 ^a^	107.0 ± 7.6 ^b^	128.9 ± 8.7 ^a^	134.4 ± 8.4 ^a^	0.23	0.002	0.003
CD14 (ng/mL)	1255.9 ± 38.0	1172.3 ± 30.8	1282.7 ± 42.6	1210.8 ± 33.9	0.0057	0.22	0.92
LBP (ng/mL)	5315.4 ± 275.9	5097.4 ± 201.6	5647.1 ± 309.7	5542.0 ± 346.8	0.42	0.03	0.78
LBP:sCD14	4.2 ± 0.2	4.4 ± 0.2	4.4 ± 0.2	4.6 ± 0.2	0.27	0.10	0.94
Endotoxin (EU/mL)	32.3 ± 2.0	30.6 ± 2.1	36.3 ± 2.5	32.5 ± 2.3	0.13	0.27	0.55

^1^ Data (means ± SE, *n* = 24) were analyzed by two-way repeated measures ANOVA. Means not sharing a common superscript are significantly different based on post-hoc analysis (*p* ≤ 0.05). ^2^ LDL-cholesterol was calculated using the Friedewald equation [34]. Abbreviations: BMI, body mass index; COMP, comparator beverage; DBP, diastolic blood pressure; GIP, glucose-dependent insulinotropic polypeptide; GLP-1, glucagon-like peptide; HDL-C, high-density lipoprotein-cholesterol; HOMA-IR, homeostasis model assessment of insulin resistance; LBP, lipopolysaccharide-binding protein; LDL-C, low-density lipoprotein-cholesterol; MEB, milk-fat-globule-membrane-enriched beverage; SBP, systolic blood pressure.

## Data Availability

Data are available from the study authors upon reasonable request.

## Supplementary Materials

The following supporting information can be downloaded at https://www.mdpi.com/article/10.3390/nu15143259/s1, Table S1: Four-day rotating menu of daily prescribed foods during the randomized controlled trial. Table S2: Average nutrient composition of 4-day 2200 kcal diet. Table S3: Human primers used for RT-qPCR gene expression.
