# Supplementary material for: Milk-Fat-Globule-Membrane-Enriched Dairy Milk Compared with a Soy-Lecithin-Enriched Beverage Did Not Adversely Affect Endotoxemia or Biomarkers of Gut Barrier Function and Cardiometabolic Risk in Adults with Metabolic Syndrome: A Randomized Controlled Crossover Trial
Source: Nutrients. 2023 Jul 23;15(14):3259. doi: 10.3390/nu15143259 (PMC10384269; doi:10.3390/nu15143259)
Supplement: Supplementary file 1 [file nutrients-15-03259-s001.zip › nutrients-2501291-supplementary.pdf]

Supplementary Table 1. Four-day rotating menu of daily prescribed foods during the randomized controlled trial.

Day 1	
	**Preparation instructions**
**Breakfast:**	Mix grits with ¾ cup of water in a bowl. Microwave for 1-1:15 min. Stir in maple syrup and raisins.
Grits	
Raisins	
Syrup	
Orange juice	
Coffee (not provided) with provided coffee creamer (optional)	
Test beverage	
**Lunch:**	Cook Turkey Burger in microwave or oven/toaster until warm. Can also cook turkey burger on pan. Make sure to fully cook. Assemble Turkey Burger on bun with provided tomato, mustard and mayonnaise.
Turkey burger on bun with condiments	
Chips	
Applesauce	
Test beverage	
**Dinner:**	Warm stew in microwave or oven/toaster oven until warm. (remove from plastic storage container and place in an oven safe bowl if warming in oven). Toast roll if desired.
Beef and potato stew	
Roll and margarine	
Test beverage	
**Snacks:**	
Graham crackers	
Fruit bowl x2	
Chips	

Day 2	
	**Preparation instructions**
**Breakfast:**	Toast bagel (optional), microwave egg and assemble bagel sandwich with egg white topped with Daiya Cheese shreds. Warm potatoes in microwave or oven/toaster oven (remove from plastic).
Bagel sandwich with egg white and daiya cheese	
Roasted potatoes	
Apple juice	
Coffee (not provided) with provided creamer (optional)	
Test beverage	
**Lunch:**	Mix Tuna Pouch, with onion, celery, and mayo found in containers. Assemble prepared tuna salad on bun.
Tuna salad on bun	
Grapes	
Cookies	
Test beverage	
**Dinner:**	Heat Pasta for 60seconds (transfer from plastic) (may make popping sound, this is normal). Microwave asparagus in microwave until warm. Chicken Meatballs given frozen. Please cook in microwave for 1min or until hot.
Pasta with chicken meatballs and marinara sauce	
Asparagus	
Test beverage	
**Snacks:**	Soft Pretzel provided frozen, please heat in oven for 3-4min at 400 °F. Alternatively, heat in microwave for 30seconds.
Fruit cup x2	
Soft pretzel with honey mustard dipping sauce	

Day 3	
	**Preparation instructions**
**Breakfast:**	Bacon is given fully cooked, ready to eat. Place in microwave for desired temperature. Warm French toast casserole in microwave or oven/toaster oven. Drizzle maple syrup and chopped dried mango over French toast casserole.
French toast casserole with dried mango and syrup	
Turkey bacon	
Cantaloupe	
Coffee (not provided) with provided creamer (optional)	
Test beverage	
**Lunch:**	Please fully cook lean ground beef in oven or skillet before eating on bun. Warm hamburger in microwave or oven/toaster oven. Assemble on bun and top with, mustard and ketchup.
Lean ground beef	
Hamburger on bun with condiments	
Grapes	
Test beverage	
**Dinner:**	Warm pork and gravy in microwave or oven/toaster oven after transferring to oven safe container. Microwave mixed vegetables until warm. Heat pasta for 60 seconds.
Pork loin on pasta with gravy	
Mixed vegetables with margarine	
Test beverage	
**Snacks:**	
Rice cake	
Fruit cup | salsa	
Larabar | Pita Chips | Twizzlers	

Day 4	
	**Preparation instructions**
**Breakfast:**	Warm muffin/bread in microwave or oven/toaster oven on plate. Enjoy with peanut butter and jelly.
Banana muffins	
Jelly	
Peanut butter	
Dried mango	
Coffee (not provided) with provided creamer (optional)	
Test beverage	
**Lunch:**	Chicken not cooked, stir fry vegetables not cooked, rice not cooked. Prepare chicken as desired, warm stir fry vegetables in frying pan or microwave. Put rice in microwave safe bowl and heat for 90 seconds, do not add water. Make sure to fully cook chicken.
Chicken and stir-fry vegetables on rice with balsamic sauce	
Test beverage	
**Dinner**	Warm Jambalaya in microwave or in an oven/toaster oven in an oven safe container. Toast bread if desired.
Shrimp and sausage jambalaya	
Test beverage	
French bread and margarine	
**Snack:**	
Clif z-bar	
Carrots	
Fruit cup	

[1]Participants were instructed to consume 3 daily test beverages with breakfast, lunch, and dinner.

Supplementary Table 2. Average nutrient composition of 4-day 2200 kcal diet

	Mean
**Macronutrients**	
Protein (g)	97.9
Carbohydrate (g)	303.8
Dietary Fiber (g)	17.5
Total fat (g)	69.7
Saturated fat (g)	25.2
**Minerals**	
Calcium (mg)	1301
Iron (mg)	15.5
Magnesium (mg)	306
Phosphorus (mg)	1467
Potassium (mg)	3428
Sodium (mg)	2075
Zinc (mg)	11.4
**Vitamins**	
Vitamin A (μg)	3375
Vitamin E (mg)	11.4
Vitamin D (mg)	12.9
Vitamin C (mg)	154.2
Thiamin (mg)	2.6
Riboflavin (mg)	2.8
Niacin (mg)	26.4
Vitamin B6 (mg)	2.4
Vitamin B12 (μg)	6.4
Choline (mg)	337.1
Vitamin K (μg)	93.6
Folate (μg)	409

[1] Values were calculated using NDSR 2022.

Supplementary Table 3. Human primers used for RT-qPCR gene expression.

Gene	Forward Primer (5’-3’)	Reverse Primer (5’-3’)
TLR4	GCTTCTTGCTGGCTGCATAA	GAAATGGAGGCACCCCTTC
TNFα	CTATCTGGGAGGGGTCTTCC	GGTTGAGGGTGTCTGAAGGA
MCP-1	GACCCCAAGCAGAAGTGGGT	GTGTCTGGGGAAAGCTAGGGG
IL-6	GTGGCTGCAGGACATGACAA	TGAGGTGCCCATGCTACATTT
p65	ATCCCATCTTTGACAATCGTGC	CTGGTCCCGTGAAATACACCTC
IL-8	CTTGGCAGCCTTCCTGATTTC	GTGGAAAGGTTTGGAGTATGTC
β-actin	AGAAAATCTGGCACCACACC	GGGGTGTTGAAGGTCTCAAA

Abbreviations: IL, interleukin; MCP-1, monocyte chemoattractant protein-1; TLR4, toll like receptor 4; TNFα, tumor necrosis factor-α;
